# What’s on the Other Side of the Gate: A Structural Perspective on DNA Gate Opening of Type IA and IIA DNA Topoisomerases

**DOI:** 10.3390/ijms24043986

**Published:** 2023-02-16

**Authors:** Vita Vidmar, Marlène Vayssières, Valérie Lamour

**Affiliations:** 1 Institut de Génétique et de Biologie Moléculaire et Cellulaire (IGBMC), Université de Strasbourg, CNRS UMR 7104, Inserm U 1258, 67400 Illkirch, France; 2 Hôpitaux Universitaires de Strasbourg, 67098 Strasbourg, France

**Keywords:** DNA topoisomerase, DNA supercoiling, DNA relaxation, DNA decatenation, type IA topoisomerase, type IIA topoisomerase, structural biology

## Abstract

DNA topoisomerases have an essential role in resolving topological problems that arise due to the double-helical structure of DNA. They can recognise DNA topology and catalyse diverse topological reactions by cutting and re-joining DNA ends. Type IA and IIA topoisomerases, which work by strand passage mechanisms, share catalytic domains for DNA binding and cleavage. Structural information has accumulated over the past decades, shedding light on the mechanisms of DNA cleavage and re-ligation. However, the structural rearrangements required for DNA-gate opening and strand transfer remain elusive, in particular for the type IA topoisomerases. In this review, we compare the structural similarities between the type IIA and type IA topoisomerases. The conformational changes that lead to the opening of the DNA-gate and strand passage, as well as allosteric regulation, are discussed, with a focus on the remaining questions about the mechanism of type IA topoisomerases.

## 1. Introduction

In all organisms, the intertwined structure of the DNA double helix requires the intervention of DNA topoisomerases in order to maintain the homeostasis of DNA topology during the cell cycle. Topoisomerases are involved in all aspects of DNA metabolism, from replication, DNA repair, and recombination, to gene expression due to their essential role in resolving topological problems [1]. This large family of enzymes can recognise different DNA topologies and catalyse a wide variety of reactions such as DNA relaxation/supercoiling, catenation/decatenation, and knotting/unknotting [2]. All topoisomerases perform the topological transactions by cleaving and resealing DNA, and this reaction involves an intermediate where the enzyme forms a covalent bond with the DNA backbone, forming a transient cleavage complex (Top-cc) [3]. Topoisomerases are divided into two types, depending on whether they introduce single-strand (type I) or double-strand breaks (type II). They are further classified into five subtypes (IA, IB, IC, IIA, or IIB) based on structural homology and reaction mechanisms [4]. Most topoisomerases (types IA, IIA, and IIB) employ a strand passage mechanism in which they transport another segment through the transient break in the DNA. Others (IB and IC) work by a mechanism of controlled rotation, where they let the free end of the cleaved DNA rotate around the intact strand.

**Figure 1:**
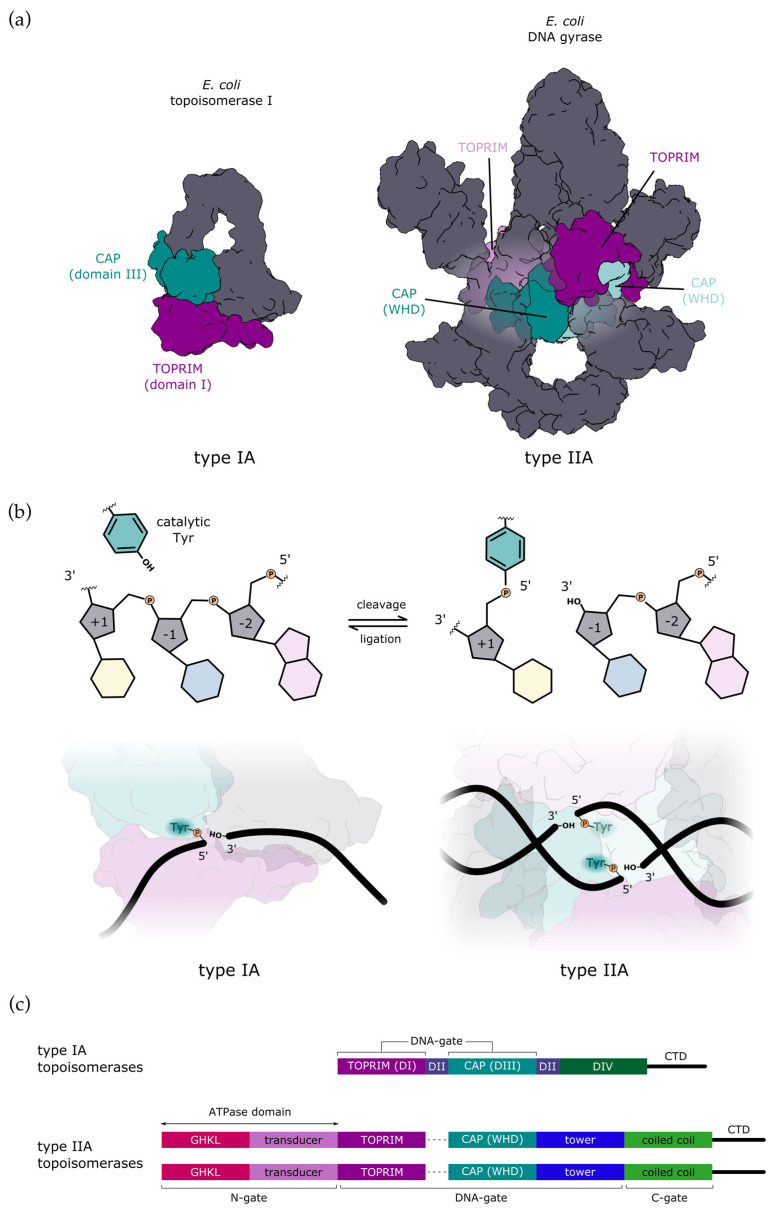
Comparison of type IA and type IIA topoisomerases. (**a**) All type A topoisomerases share catalytic domains for DNA binding and cleavage: CAP domain (dark cyan) bearing the catalytic tyrosine and TOPRIM domain (dark magenta) with a cluster of conserved acidic residues that bind Mg^2+^ which is also required for catalysis. Type IA topoisomerases are monomers with one CAP and one TOPRIM domain, whereas type IIA topoisomerases are dimeric and thus have two pairs of catalytic domains in trans (the second pair is shown in a lighter colour). *E. coli* topoisomerase I is shown without CTD for clarity. (**b**) During DNA cleavage, catalytic tyrosine forms a covalent bond with the 5′-phosphate of the DNA backbone in both types of topoisomerases (top). The reaction is reversible, with the equilibrium shifting toward ligation. Type IA topoisomerases cleave single-stranded DNA (bottom left), whereas type IIA topoisomerases introduce a transient double-strand break with a stagger of 4 bp (bottom right). (**c**) Schematic representation of domain organisation. Type IA normally consists of a single polypeptide chain encoding four topoisomerase domains, here labelled as DI to DIV. Additionally, they may have a variable CTD. Type IIA topoisomerases are dimers with two copies of each domain. In bacteria, they are heterotetramers (A_2_B_2_), whereas in eukaryotes, they are homodimers (A_2_), where each half of the enzyme is fused into a single subunit (denoted with a dashed line).

The first topoisomerases were identified and characterised in the 1970s as the bacterial ω protein [5] and DNA gyrase [6], which were later classified as type IA and type IIA topoisomerases (Figure 1a). DNA relaxation is one of the major activities of type IA and IIA topoisomerases. However, the bacterial type IIA DNA gyrase and type IA reverse gyrase have the unique ability to introduce negative or positive supercoils, respectively [2]. The type IA topoisomerases relax negatively supercoiled DNA in an ATP-independent manner, whereas the type IIA topoisomerases relax negatively and positively supercoiled DNA in the presence of ATP [2].

The type IA enzymes act on a larger variety of substrates, such as ssDNA, nicked DNA, dsDNA, and RNA, when compared to the type IIA enzymes, which carry out topological transactions solely on dsDNA. In addition to the relaxation activity of supercoils, some topoisomerases preferentially catenate/decatenate, or knot/unknot DNA molecules [2]. For these particular activities, the substrates of the bacterial Topo I enzymes are restricted to ssDNA or nicked DNA. TopIII, another type of IA enzyme, can relax and decatenate DNA and RNA [7,8]. In bacteria and human cells, the types IIA, TopoIV, and TopoIIα, respectively, also possess a robust decatenation activity [2,9,10].

The mechanism of DNA cleavage involves a conserved catalytic Tyr in the active site that participates in a reversible transesterification reaction, forming a transient phosphotyrosine bond with the 5′ ends of the DNA backbone (Figure 1b) [3]. The monomeric structure of the type IA topoisomerase and the dimeric structure of the type IIA or IIB topoisomerases condition the introduction of single- and double-strand breaks respectively (Figure 1b). The mechanism of single- or double-stranded passage that follows the cleavage of a first DNA molecule is tightly regulated to prevent the accumulation of DNA breaks that can lead to cell death.

Structural investigations of the catalytic cycle of the type IA and type IIA enzymes have provided most of the molecular details of the DNA cleavage/re-ligation mechanism and of the conformations that lead to DNA strand passage and DNA relaxation. In particular, the common structural organisation of the cleavage site of the IA and IIA enzymes has been revealed by x-ray crystallography [4]. They contain a common set of CAP domains adopting a Winged-Helix Domain fold (WHD) comprising the conserved tyrosine and a TOPRIM domain with acidic residues chelating two magnesium ions (Figure 1a) [11]. Although the IIB enzymes act with a strand passage mechanism, their primary sequence and global structural organization, down to the CAP and TOPRIM catalytic domains, differ from the IIA enzymes [12,13,14,15,16]. Recent studies have shown that decatenation or unknotting are the preferred activities of the bacterial type IIB topoisomerase VI, which may be related to the fact that they work specifically on certain topologies [17].

In comparison to type IA enzymes, structural studies of type IIA topoisomerases have revealed molecular details for many conformations of the DNA binding/cleavage domain, specifically the closed, partially opened, or fully opened DNA-gate. While structural information is now available to understand how DNA cleavage/re-ligation is catalysed by type IA enzymes, the mechanism of DNA transport through the break and how the structural domains contribute to the opening of the DNA gate at the molecular level remains quite elusive for the type IA family. In this review, we present an overview of the conformational states that condition DNA relaxation and strand passage, focusing on the DNA-gate of the type IA and IIA enzymes, with an emphasis on the remaining questions pertaining to the mechanism and regulation of gate opening in the type IA family.

## 2. Mechanism of DNA-Gate Opening by Type IIA DNA Topoisomerases

Type IIA topoisomerases are present as dimeric (eukaryotes) or heterotetrameric (prokaryotes and archaea) assemblies (Figure 1c). They contain two copies of evolutionarily conserved domains forming three protein interfaces, also called “gates”. The ATPase (GHKL) domains form the N-gate and are responsible for ATP hydrolysis [18]. The DNA gate is composed of the conserved CAP (WHD), TOPRIM, and Tower domains (Figure 1c). They contain the conserved catalytic tyrosine and acidic residues chelating two magnesium ions and are responsible for DNA cleavage [19,20,21]. The C-gate is composed of two long coil-coiled helices at the C-terminal end of the protein, where a DNA duplex can exit the enzyme interface after strand passage [22,23,24,25]. Together, biochemical and structural data have led to the proposal of a “three-gates” mechanism where the protein interfaces successively interact with DNA duplexes at the different steps of the intermolecular reaction of DNA relaxation, catenation, or unknotting [18,19,21,22,23,24,25,26,27,28,29]. Interestingly, the type IIB family, for which structures of the archaeal TopoVI and eukaryotic Spo11 have been solved, lacks the C-gate, and only structures of the closed conformation of the DNA gate have been determined so far [14,16].

### 2.1. Structural Rearrangements Leading to DNA-Gate Opening in Type IIA Topoisomerases

**Figure 2:**
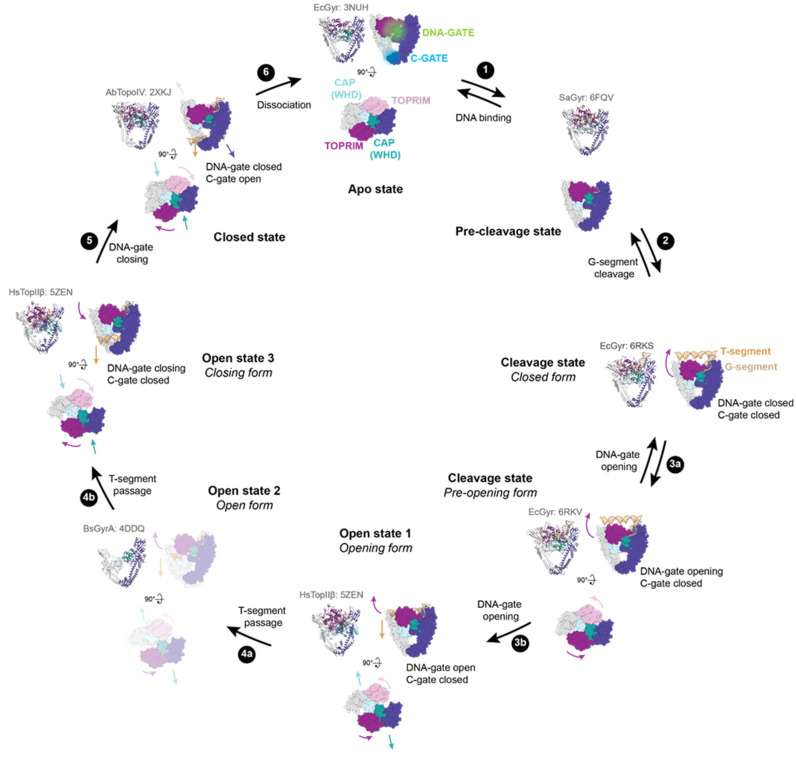
DNA-gate opening mechanism of type IIA enzymes. All the shown intermediates were structurally determined by X-ray crystallography or cryo-electron microscopy (cryo-EM). PDB accession codes are indicated in light grey. Structure representations restricted to the DNA-binding/cleavage domain were generated from the *E. coli* DNA gyrase structure without DNA and the insertion domain for clarity. TOPRIM domains are coloured dark magenta and plum. CAP (WHD) domains are coloured light blue and dark cyan. The other domains are coloured in dark slate blue and grey. The G-segment is shown in tan, and the T-segment (modelled) is in sandy brown. (1) G-segment binds to the DNA binding-groove formed by the TOPRIM-CAP(WHD)-Tower domains. (2) DNA binding triggers a rotation of the TOPRIM domains, resulting in a repositioning of the acidic motif with metal ions close to the catalytic tyrosine residues (CAP/WHD) and cleavage of the G-segment. (3) DNA-gate opening involving sliding and swivelling motions (TOPRIM/WHD (CAP)) of the two halves of the protein against each other. 3a and 3b show the transition to the closed form to the pre-opening form of the DNA-gate. The dimer remains together thanks to interactions in the C-gate. (4) From these movements, a funnel-shaped channel is revealed between the two halves of the DNA-gate allowing T-segment passage to change DNA topology. 4a shows the T-segment passage through the upper part of the DNA-gate. The structure of GyrA in an opened conformation suggests that a wider opening of the DNA gate happens to complete strand passage as illustrated in step 4b. (5) After the T-segment passage through cleaved G-segment, the DNA-gate is closed and the G-segment is re-ligated. Once the DNA-gate is closed, the C-gate opens to release the T-segment. (6) After a complete reaction cycle, the enzyme dissociates from the DNA and is reset for another cycle.

The first step of the catalytic cycle is the binding of DNA, the G-segment (for “Gated” DNA), at the dimeric interface of the DNA-gate. Crystal structures of the DNA-binding/cleavage domain revealed conformational changes of the DNA-gate upon DNA binding [19,24,26,30] (Figure 2, step 1). The G-segment binding triggers a ~80° rotation of the TOPRIM domains relative to their initial position, leading to new protein-protein interactions with the CAP and Tower domains from the opposite monomer. This movement creates a positively charged groove in which the double helix of DNA can perfectly fit [24]. Cleavage of DNA is mediated by two metal ions for type IA and type IIA topoisomerases [11] (Figure 2, step 2). After DNA binding, the metal ion binding motif (E-DxD) in the TOPRIM domain is positioned close to the conserved catalytic tyrosine in the CAP domain. The comparison of the active site structures with cleaved or uncleaved DNA has revealed finely regulated conformational changes before, during, and after DNA cleavage [11,25,31,32,33,34,35].

The overall comparison of DNA-gate structures in presence of cleaved or uncleaved DNA suggests that protein interfaces move when they open or close. These movements are carefully regulated to prevent DNA breaks in the genome. ATP hydrolysis at the N-gate triggers allosteric signals controlling the opening or closing of the gates, notably through the positioning of the catalytic tyrosine residues [11,24]. Therefore, once the G-segment is cleaved, the C-gate is kept closed so that the DNA gate can open for the transport of another DNA duplex, called the T-segment (Figure 2, steps 3 and 4). When the G-segment is re-ligated, the DNA-gate closes and the C-gate opens, allowing the T-segment to exit (Figure 2, step 5). The fine regulation of the protein interfaces opening during DNA cleavage is of paramount importance for the unidirectionality of DNA transport (Figure 2, step 4).

Type IIA topoisomerases also modify the DNA geometry in order to simplify DNA topology. DNA bending is induced by a conserved isoleucine (Ile 833 for yeast TopII) that intercalates between two base pairs [24]. The high degree of DNA bending generated by Topo IIA may explain its preferential binding to highly bent and flexible DNA regions [36,37].

The first observation of type IIA DNA-gate structures in closed conformation reported twenty years ago led to the proposal that DNA-gate opening is similar to a book-opening movement [19,26]. However, a crystal structure of the DNA-gate in an open conformation has been published recently, and a new opening mechanism has been proposed [34] involving a path for the T-segment through the gate as well as conformational changes that could account for the transition from a closed to an opened state. In this conformation, the dimer is maintained only by protein interactions at the C-gate. The CAP domains move away to create a funnel-shaped channel between the two parts of DNA-gate. This cavity is able to accommodate the T-segment in the DNA-gate. The helical axes of the two parts of the G-segment are shifted ~20° and move in the opposite direction when compared to the initial axis of the G-segment. Therefore, a rocker-switch-type movement of the CAP domains is responsible for DNA-gate opening (Figure 2, step 3b).

The cavity created between the two subunits is increased by local conformational changes that involve an outward movement of the TOPRIM and Tower domains surrounding the G-segment. Separation of the two parts of the G-segment creates a channel in the DNA-gate that can accommodate a B-form DNA duplex (Figure 2, step 4a). Consequently, DNA-gate opening relies on an outward tilting of the two halves of DNA-gate and a sliding/swivelling of the two subunits against each other, breaking the G-segment axis. However, the cavity at the bottom of the funnel is narrower than the DNA duplex. It is therefore necessary to further expand this region for strand passage. The open conformation structure observed in structural studies thus far [34] corresponds to an early intermediate of T-segment passage through DNA-gate. Recent evidence from cryo-EM structures of full-length DNA gyrase and a human type IIA topoisomerase in closed and pre-opening states of the DNA-gate supports the “sliding and swivelling” opening mechanism [38,39]. The pre-opening conformation precedes the open conformation, where the G-segment is still tightly bound to the groove at the DNA-gate interface but the CAP, TOPRIM, and Tower domains have started to undergo conformational changes.

### 2.2. Regulation of DNA Gate Opening of Type IIA Topoisomerases

DNA-gate structures solved over the past decades have helped to unravel the structural mechanism of type IIA DNA-gate opening. However, many questions remain unanswered, particularly about communication between functional domains in the presence of DNA crossovers, as well as the molecular basis for distinguishing specific DNA topology and the role of the variable C-terminal domain (CTD) in catalytic cycle regulation. In particular, the eukaryotic topoisomerase II possesses a non-conserved CTD, which is predicted to be unfolded and connected to the last helix of the coiled-coil domain. Until now, no structural information is available for this region. The CTD contains a nuclear localization sequence and several post-translational modifications (PTM) [40]. It has been proposed that the PTM may modulate the activity of human topoisomerase IIα and the recruitment of associated proteins [40,41]. The recent cryo-EM structure of human topoisomerase IIα has revealed that the linker connecting to the CTD stimulates DNA cleavage and enhances the catalytic activity of human TopIIα [39]. The CTD of DNA gyrase from different bacterial species, on the other hand, is conserved and adopts a β-pinwheel shape [42,43,44,45,46]. DNA wrapping around this domain is critical for the supercoiling activity of DNA gyrase [43,47]. Topoisomerase IV, the other type of IIA bacterial topoisomerase, possesses a degenerate form of the homologous GyrA CTD [45,46]. This structural difference accounts in part for the distinct supercoiling and decatenation activities of DNA gyrase and TopIV. Indeed, the deletion of DNA gyrase CTD converts it into a TopIV enzyme [47]. In addition, the deletion of the TopIV CTD results in a loss of activity on positively supercoiled DNA or catenanes [45]. An acidic tail containing a stretch of non-conserved negatively charged residues is another structural element typical of *E. coli* DNA gyrase CTD. It has been suggested that this extension may tightly regulate the supercoiling activity of the enzyme through interaction with the non-conserved domain insertion found in the TOPRIM domain of *E. coli* DNA gyrase [48,49,50]. All together, the functional domains of topoisomerase IIA communicate through allosteric signals, which result in the opening and closing of the three gates to change the chirality of DNA duplex supercoiling.

## 3. Mechanism of DNA Gate Opening by Type IA DNA Topoisomerases

Although there are some mechanistic parallels with type IIA enzymes, the catalytic cycle of type IA topoisomerases is more poorly characterised. While some steps of the catalytic cycle are firmly established with solid structural and biochemical data, the mechanisms of gate-opening and strand passage steps remain elusive, and the reaction intermediates are still largely hypothetical due to their transient nature.

**Figure 3:**
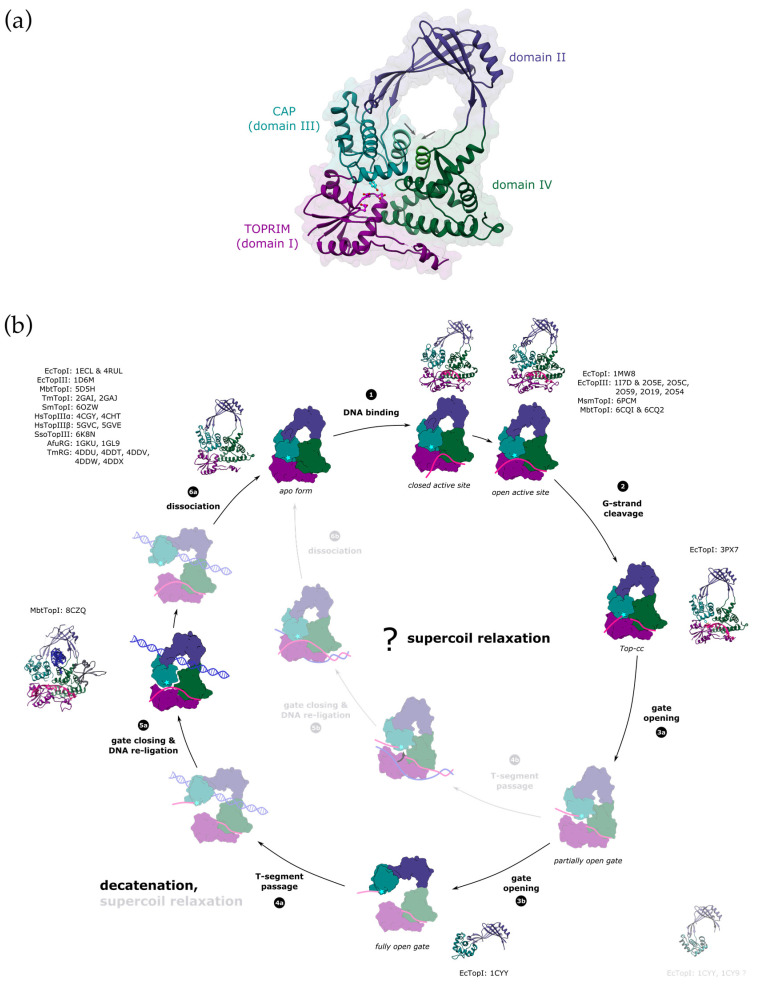
Proposed reaction mechanism of type IA enzymes. (**a**) Domain organisation of type IA topoisomerases. Type IA topoisomerases consist of four domains (labelled I–IV). CAP and TOPRIM domains contain the active site (the catalytic Tyr is shown in cyan and the residues from the TOPRIM motif are shown in magenta), and they also form the DNA-gate. The interface alpha-helices in domains DIII and DIV that are closing the entrance into the toroid hole are labelled with arrows. PDB ID: 1ECL. (**b**) Topo IA catalytic cycle. Structurally characterised intermediates are shown in full colour with the crystal structures and the PDB accession codes next to them (EcTopI = *Escherichia coli* topoisomerase I, EcTopIII = *Escherichia coli* topoisomerase III, MbtTopI = *Mycobacterium tuberculosis* topoisomerase I, TmTopI = *Thermotoga maritima* topoisomerase I, SmTopI = *Streptococcus mutans* topoisomerase I, HsTopIIIα = human topoisomerase IIIα, HsTopIIIβ= human topoisomerase IIIβ, SsoTopIII = *Sulfolobus solfataricus* topoisomerase III, AfuRG = *Archaeoglobus fulgidus* reverse gyrase, TmRG = *Thermotoga maritima* reverse gyrase, MsmTopI = *Mycobacterium smegmatis* topoisomerase I). Domains I to IV are coloured in dark magenta, dark slate blue, dark cyan, and dark green. The catalytic tyrosine is marked with a cyan star. G-strand is shown in deep pink and the T-segment in blue (either single-stranded or double-stranded). First, the single-stranded G-segment binds to the DNA-binding groove that runs along domain IV. This triggers a conformational change in the enzyme, which results in the active site rearrangement and aligns the scissile bond of the DNA for cleavage (1). Transesterification reaction leads to the formation of the covalent complex (Top-cc), where the 5′-end of the cleaved DNA is covalently bound to the catalytic tyrosine. The 3′-end remains tightly bound within the DNA-binding groove (2). The gate located between the domains I and III opens to form a gap in the G-segment to allow for the strand passage (3). The gate opening might proceed through several mechanistic/kinetic steps [52]. The process could begin with domain sliding that separates the domain III from TOPRIM but retains its interaction with the domain IV (3a, partially open gate) [53]. Breaking the contact with domain IV would result in the opening of the toroidal hole (3b, fully open gate). The T-segment must pass through the nicked G-strand to change the DNA topology. The enzyme is believed to capture the T-segment within the central hole, which is capable of accommodating dsDNA [54,55]. The binding of a double-stranded T-segment is likely accompanied by a conformational change that results in widening of the toroid hole [56] (4a). Alternatively, partial DNA-gate opening might already provide an opening that is big enough for passage of single-stranded DNA, leading to supercoil relaxation [53] (4b). Following the T-segment passage, the gate must close to bring the broken DNA ends back together, and the nick gets re-ligated (5a,b). This process could be potentially facilitated by rewinding the duplex [57]. After the complete reaction cycle, the enzyme dissociates from the DNA (6a,b). The gate would have to re-open to release the T-segment from the toroidal hole. Both supercoil relaxation and decatenation could happen either way, but path 4a→6a with a fully open gate would favour passage of double-stranded segments (decatenation), and the path 4b→6b with a partially open gate the transfer of single-stranded segments (supercoil relaxation).

The catalytic core of type IA topoisomerases consists of four structural domains (I–IV) arranged in a padlock shape with a distinct toroidal hole in the centre [51] (Figure 3a). The CAP (DIII) and TOPRIM (DI) domains work together to form a single DNA-gate for strand passage. The CAP domain is flexibly connected to the rest of the enzyme via domain DII, which mediates gate opening. Domain DIV forms a binding site for the G-strand.

### 3.1. Structural Mechanisms Leading to DNA-Gate Opening in Type IA Topoisomerases

Type IA enzymes can act on a wide variety of substrates, including RNA, but have a strict requirement for single-stranded regions. In the apo form, the active site of type IA topoisomerase is buried in the interface between the CAP (DIII) and TOPRIM (DI) domains [51,58,59]. The binding of ssDNA (G-segment) to the DNA-binding groove, which is running along the domain DIV, induces a domain rearrangement, which leads to the formation of the catalytically competent active site and aligns the scissile phosphodiester bond for cleavage [60,61,62] (Figure 3b, states 1 and 2). Although the G-strand is held in the DNA binding groove mostly via interactions with the DNA backbone [62,63,64], type IA topoisomerases usually display DNA sequence selectivity for cleavage [65,66]. A cytosine residue at position −four relatives to the cleavage site is required for the bacterial topoisomerase IA enzymes. A recent genome-wide study identified a consensus binding and cleavage motif for *E. coli* topoisomerase I, whose sequence is AT-rich and contains a strictly conserved cytosine residue [67]. A crystal structure of *E. coli* topoisomerase I, a covalent intermediate, revealed that the enzyme has a cavity that specifically accommodates this base [68] and acts as a molecular ruler that helps to precisely position the scissile bond in the active site [65]. Interestingly, it was shown that for human topoisomerase IIIβ, the sequence requirements for RNA cleavage significantly differ from the DNA cleavage sequence [66]. To date, there is no structure of type IA topoisomerase bound to RNA that would explain the molecular basis for the differential selectivity. However, it might be a consequence of different backbone conformations of DNA vs. RNA and thus differences in their interactions with the enzyme.

After the nucleophilic attack by the active site tyrosine residue, the G-strand gets cleaved, leaving its 5′-end covalently bound to the enzyme, whereas the free 3′-end remains non-covalently associated with domain DIV (Figure 1b, state 2). After the strand passage event, the DNA is re-ligated in the reversible reaction, where the free 3′-OH group of the DNA acts as a nucleophile. For this transesterification reaction, divalent cations are required (usually magnesium), which are coordinated by conserved residues of the TOPRIM motif. Based on the evidence for the type IIA topoisomerases, a common two-metal ion mechanism was proposed [11] in which the first metal ion stabilises the transition state, whereas the second is believed to participate in DNA binding. Although type IA has homologous catalytic domains with conserved residues [69], their mechanisms might be slightly different. It is not entirely clear if cleavage needs a metal ion or not, because type IA topoisomerases have an extra lysine residue in the active site that could replace the cation in the cleavage reaction [11]. Re-ligation, on the other hand, strictly depends on the metal ions, which are needed to precisely align the DNA ends for re-joining. In those few crystal structures where the ion is bound to the active site, only one could be identified [61,70,71], making it uncertain whether one ion is sufficient for the reaction or whether there should be two ions, like in type IIA topoisomerases, with the second metal ion bound only transiently.

While the single-strandedness of the G-segment is an absolute requirement for cleavage, there seems to be almost no limitation for the nature of the T-segment. Type IA topoisomerases are capable of performing a wide range of topological reactions: they can relax (negative) supercoils [5], decatenate hemicatenated as well as fully catenated substrates [72], and untangle RNA [73,74]. For supercoil relaxation and manipulation of RNA topology, transfer of a single strand generally needs to occur. However, decatenation of fully catenated substrates requires several catalytic cycles (strand passage steps) and involves hemi-catenated intermediates. A topoisomerase can decatenate two DNA molecules either in two steps, separately transferring a whole duplex through two single-stranded nicks, or in four steps, passing only one strand at a time (two strands through two gaps) [75,76]. Moreover, it was shown that plasmids containing R-loops are excellent substrates for supercoil relaxation by bacterial TopI [77]. Although not formally demonstrated, this implies that the topoisomerase could also use a DNA/RNA hybrid as the T-segment. All this raises the question of how one enzyme can carry out so many topological reactions.

Three decades ago, when the first crystal structure of the type IA topoisomerase (the catalytic core of the *E. coli* TopI) was solved, an elegant mechanism unifying all these ‘complicated’ strand transfers was proposed: after cleavage, the toroid structure of the enzyme opens up and transiently captures the T-segment inside its central cavity [51]. The enzyme’s clamp-like shape would allow it to be opened, and the size of the toroid hole in the closed state is approximately 25 Å, which is large enough to accommodate either single-stranded or double-stranded DNA (a diameter ~ 20 Å). Additionally, the inner side of the hole is lined with positively charged residues, which would enable non-specific DNA binding via electrostatic interactions with its backbone. It was later shown that both single-stranded and double-stranded DNA can be captured inside the central hole by two DNA decatenating enzymes, *E. coli* TopIII [54] and *M. smegmatis* TopI [55]. Each group used a different approach to stabilise the topoisomerase in the closed state after the DNA capture. Li et al. created a disulfide bond between domains DIII (CAP) and DIV to keep the central cavity closed, whereas Leelaram et al. used an inhibitory antibody to keep the topoisomerase in the closed state. Both have also shown that preventing the topoisomerase from opening inhibits its catalytic activity (DNA relaxation). However, there are two considerations with these experiments. First, the capture of DNA inside the central hole was not a result of the topoisomerase reaction (e.g., a trapped intermediate of the DNA relaxation), since for their DNA-trapping experiments both groups used a catalytically dead enzyme, where the catalytic tyrosine was mutated into phenylalanine. Although this undisputedly proves that the toroidal hole can open and accept a DNA molecule, it raises the question as to whether this is a part of the catalytic cycle or just a result of stochastic DNA trapping by the opening and closing of the enzyme in solution. In that regard, it was shown that topoisomerases are very flexible enzymes that readily undergo conformational changes even in the absence of DNA [78]. Second, the stabilisation of the enzyme in the closed conformation might not only affect gate opening but also other steps in the catalytic cycle. The inherent flexibility of topoisomerases is crucial for their function, since mutations or factors that impair conformational changes also affect earlier stages of the catalytic cycle, e.g., DNA binding or cleavage [55,79], which makes it very difficult to uncouple their effects from the subsequent steps.

A crystal structure of *M. tuberculosis* TopI bound to the G-segment and the DNA trapped within the toroid hole was recently published and proposed to represent the state immediately following the G-strand re-ligation [56] (Figure 3b, state 5a). However, since only one DNA oligonucleotide was used to form the complex, the trapped T-segment is a non-canonical parallel duplex that formed as a result of crystal packing. In this complex, TopI only interacts with one of the strands, making sequence-independent contacts with the DNA backbone, which would be consistent with the T-segment being either single- or double-stranded.

**Figure 4:**
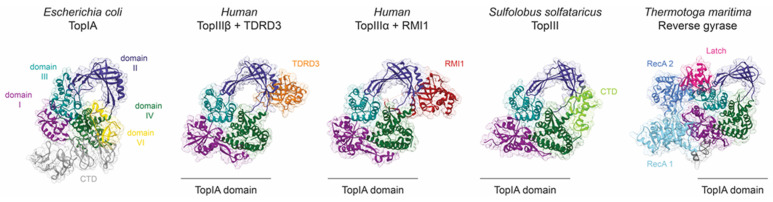
Structural elements, partners, or associated domains regulate topoisomerase IA activity. The domains I to IV of topoisomerase IA are coloured dark magenta, dark slate blue, dark cyan, and dark green. Additional domains of *E. coli* TopI (PDB 4RUL) located at the C-terminal end are coloured grey, and the Zn finger of domain VI interacting with domain DII appears in yellow. The catalytic domain of topoisomerase III and reverse gyrase adopts the typical topoisomerase IA toroidal fold. TDRD3 (orange) or RMI1 (red) are accessory proteins interacting mainly with the toroidal region of human TopIIIβ (PDB 5GVE) and TopIIIα (PDB 4CGY), respectively. Similarly, the C-terminal domain (CTD in light green) of TopIII from *S. solfataricus* (PDB 6KN8) interacts with the toroid domain. Reverse gyrase (PDB 4DDU) is composed of two RecA helicases domains (light and medium blue) separated from the Topo IA domain by the latch domain (deep pink).

Another question pertaining to the generally accepted hypothesis on the reaction mechanism is whether all type IA topoisomerases can bind DNA within their central hole. Reverse gyrases appear to have a smaller toroid hole than the other topoisomerases (~16 Å vs. ~25 Å), which can only hold single-stranded DNA but not a double helix [80,81] (Figure 4). Admittedly, this could be in line with their specialisation in positively supercoiled DNA, which would not require strand passage of the duplex. It was also suggested that a smaller hole size could contribute to the thermostability of the enzyme [80]. In type IA enzymes, the amino acid sequence of domain DII, which comprises the largest part of the toroid hole, is less conserved compared to the rest of the catalytic core [82]. Even though they generally do have positively charged residues facing the inner side of the central cavity, their number and distribution vary depending on species and subtypes [56,82]. This points to the absence of specific protein-DNA interactions within the hole. Accordingly, it was proposed that the DNA, which is trapped inside the central hole, could slide [54,83]. The lack of strong interactions would presumably facilitate rapid strand transfer into and/or out of the hole. Something similar has been proposed for the T-segment passage through the DNA-gate of type IIA DNA gyrase, except that the residues interacting with the DNA are well-conserved in this case [84]. In addition, some topoisomerases have loops inserted at different positions in the domain DII that are protruding into the hole [59,85], or present surfaces that can bind to interaction partners [70,71]. Both would result, at least to some degree, in a reduction of the effective size of the central cavity. Currently, it is unknown what the roles of these loops are, but being flexible elements, they might regulate the strand passage and/or gate opening [56,85]. The available structural as well as biophysical data suggested that type IA enzymes have sufficient plasticity to widen the central hole to accept double-stranded DNA [56,83].

Over a dozen full-length or partial crystal structures of IA topoisomerases from various organisms with or without bound DNA have been solved to date, but these structures have not provided information on the conformational changes that open the DNA-gate and promote T-segment passage [51,56,58,59,60,61,62,63,64,68,70,71,80,81,85,86]. One exception might be the structure of the isolated fragment of the DII/DIII domains from *E. coli* TopI [87] (Figure 3b, state 3b). This fragment adopts a conformation where a ‘break’ in the β-sheet of domain DII would move domain DIII away from the domains DI and DIV, thus forming an entrance into the central hole of the enzyme. The size of the opening would be sufficient to allow the passage of the DNA duplex. The structure revealed the conformational flexibility of domain DII, which is believed to mediate gate opening. However, considerations of the conformation of this fragment alone do not account for the interactions with the rest of the protein. It is worth pointing out that in the same asymmetric unit of the crystal structure, the DII/DIII fragment can be found in another conformation, which would be incompatible with the context of the full-length TopI due to a major clash with domain DIV (Figure 3b, bottom right).

Some very useful insights on DNA-gate opening come from single-molecule experiments that compared two of the most studied IA topoisomerases from *E. coli*: TopI, as an example of an enzyme that excels in supercoil relaxation but is less efficient at decatenation, and TopIII, which has the opposite properties. As it turns out, the catalytic activity of TopIII outperforms that of TopI in both DNA relaxation and decatenation. The only thing that makes TopI a better DNA-relaxing topoisomerase is a shorter waiting time between the relaxation cycles [76,88].

Mills et al. directly measured the kinetic parameters of gate opening for *E. coli* TopI and TopIII using magnetic tweezers [52]. The authors measured the distance between the DIII and TOPRIM domains as the DNA-gate opened by monitoring the DNA extension caused by DNA cleavage, which directly corresponds to the distance between the cleaved DNA ends. The measured distance was the same for both enzymes, but, interestingly, it was shown that the gate remained open much longer for TopIII than for TopI. Apart from the differences in their C-terminal domains, TopIII possesses a small insertion in domain IV, called the decatenation loop. The second difference lies in the sequence of the hinges that connect domain DII with domains DIII and DIV [89]. The molecular dynamics simulation that Mills et al. also performed suggested that, in the open conformation, the decatenation loop would make stabilising contacts with another loop in domain DII, both loops being missing in TopI [52]. This observation and the impact of the hinge sequences on the mechanism remain to be experimentally confirmed. This study also proposed that gate opening might proceed through several mechanistic/kinetic steps.

Bakx et al. performed a similar study on the human TopIIIα-RMI1-RMI2 complex, which has a primary decatenating activity, and on the *E. coli* TopI [83]. Although not at the single-molecule level, they simultaneously monitored DNA-gate opening by measuring the extension of the DNA caused by cleavage and the binding of topoisomerase to the G- and T-segments during DNA catenation, using a combination of dual-trap optical tweezers and fluorescence microscopy. Consistently, they detected a two-step change associated with DNA-gate opening, where they interpreted the second step as a widening of the gate. Furthermore, they have shown that transfer of double-stranded T-segment results in the expansion of the DNA-gate, whereas a single-stranded T-strand did not affect the gate size.

A different single-molecule study by Gunn et al. [53] monitored the relaxation of supercoils by *E. coli* TopI using magnetic tweezers, while simultaneously observing the concomitant conformational change of the protein by Total Internal Reflection Fluorescence (TIRF) Microscopy. They obtained seemingly contradictory conclusions about the topoisomerase mechanism. First of all, they proposed that domain DIII might not move away from the main body of the enzyme but instead slide closer to create a gate and capture the passing strand. Such movement could potentially be possible, but it would not result in the opening of the central hole because this would require the complete separation of domain DIII. As previously stated, two α-helices, one from domain DIII and one from DIV, form a major interface between these domains, keeping the toroid hole closed (Figure 3a). One possible explanation is that the DNA-gate only partially opens, disrupting contacts between domains DIII (CAP) and DI (TOPRIM) but not with DIV. Furthermore, Gunn et al. [53] suggested that the topoisomerase I catalytic cycle involves a sub-cycle in which the enzyme is trying to capture the T-strand, moving between closed and open conformations, with only successful events resulting in strand passage and DNA relaxation.

What could be the cause of the discrepancy between the studies by Mills et al. [52] and Gunn et al. [53], and where do their conclusions meet? One obvious answer is the experimental setup. By applying high forces on the DNA, Mills et al. likely measured the largest extent of the gate opening, whereas the assay by Gunn et al., which was monitoring supercoil relaxation, was probably closer to the native conditions in terms of the forces that act on the DNA, thus complementing the experiments by the former. Mills et al. observed that it was difficult to capture TopI in the open state due to its fast closing rate, and the interpretation of the results by Gunn et al. points in the same direction: that the gate of TopI does not readily open, but in spite of this ‘shortcoming’, it still efficiently relaxes supercoils. It was also noted by Bakx et al. that TopI does not efficiently bind and thus transfer double-stranded T-segments, which could be potentially related to the DNA-gate opening [83]. In contrast, Topo III enzymes open the gate much more easily, which would be favourable for the passage of more bulky substrates during decatenation.

Why would such discrimination between the relaxation of supercoils and decatenation be necessary? What role does the T-segment have in the catalytic cycle other than being transported? Unlike their big siblings, type IIA enzymes, the type IA topoisomerases are not powered by ATP hydrolysis, and they depend entirely on the energy stored in the DNA to drive strand passage. While the passage is unidirectional in type IIA enzymes, type IA enzymes can, in principle, transfer the strand in both directions. TopIII from *Sulfolobus solfataricus*, a hyperthermophilic archaeon, can perform two topologically opposite reactions [90]. At low temperatures, it can link single-stranded circles into double-stranded DNA (increasing the linking number of the substrate), whereas at low temperatures, it produces single-stranded circles (decreasing the linking number). Apparently, this enzyme can perform the strand passage reaction in either direction, which is determined by the temperature. Additionally, *E. coli* TopI, which normally relaxes negative supercoils, can also relax positive supercoils provided that the substrate contains a single-stranded region (e.g., a mismatched bubble or a bulge) [91]. Such substrates are often used in single molecule studies to constrain the topoisomerase to a single region on the DNA [53,88,92]. Strand passage in IA topoisomerases seems to work well in either direction, but this raises another question: given the asymmetry of the enzyme, are both directions mechanistically equivalent, or is one direction preferred over the other? Not limited exclusively to supercoiling, it appears that the geometry of the DNA matters for different IA topoisomerases. Single-molecule experiments on *E. coli* TopI and TopIII comparing supercoil relaxation and decatenation indicated that TopIII might be particularly sensitive to the type of substrate, making it inefficient for relaxing supercoils but good at decatenation, particularly with large DNA crossover angles [76,88].

The transferred segment might have more than just a passive role in the catalytic cycle—a possibility that has not been explored yet. A study on *Sulfolobus solfataricus* TopIII showed that annealing of the complementary strand facilitated re-ligation of the cleaved strand [57]. If this finding could be generally applied to other type IA topoisomerases, this would have important consequences for negative supercoil relaxation. Apart from shifting the reaction equilibrium towards re-ligation, it might also contribute to DNA rewinding after strand passage and a faster turnover of the enzyme through the different affinities for double-stranded versus single-stranded DNA. Indeed, in the crystal structures of the topoisomerases bound to the G-strand, the DNA is in a B-like conformation, which could presumably prepare it for duplex formation [62,63,64].

Taken together, it has become clear that different types of IA enzymes work in different ways. Relatively subtle structural differences result in strong preferences for different reactions (e.g., supercoil relaxation versus decatenation), although it is difficult to precisely pinpoint the molecular basis for this specificity. One possibility is that decatenation and relaxation of supercoils by the type IA enzymes might use two slightly different pathways, which are not mutually exclusive (Figure 3b). The DNA-gate must be opened for T-strand passage after the initial steps of G-segment binding and cleavage. Even though the conformational changes are mostly made up, it seems likely that there are steps in between. As a result, the DNA-gate may open to a greater or lesser extent, though it is unclear whether this necessarily entails opening the central hole. Regardless, the size of the gap would determine if the T-segment could be transported, and single-stranded segments might be able to slip through the gate before it is fully open. This would explain the observed differences, but we need more experimental evidence at this point.

### 3.2. Structural Regulation of DNA Gate Opening and Strand Passage in Type IA Topoisomerases

Although in vitro data show that the DNA-gate can open spontaneously in solution regardless of the enzymatic activity [54,55], gate opening during the catalytic cycle must be coordinated with the cleavage and strand passage steps. To improve their catalytic efficiency, type IA enzymes often have ‘helpers’, either as additional domains in the enzyme itself or through other proteins that associate with them.

The type IA topoisomerases greatly differ in their CTDs, which often contain a variable number of Zn fingers [93] or some other type of DNA-binding domain [59]. The eukaryotic Topo III enzymes additionally have RGG domains for RNA-binding [94]. These domains on the C-terminus bind DNA, usually with high affinity [62,95], forming additional stabilising interactions with the substrate that enhance the activity of the core enzyme [66,96]. Though the exact role of CTD is unknown, it is essential for catalytic activity in some topoisomerases but not in others, and its deletion only impairs enzyme processivity [59,66,86,96,97,98,99]. For example, *E. coli* and mycobacterial TopI absolutely require the CTD for their DNA relaxation activity, and it was proposed that the CTD binds the T-strand to promote its passage [62,85]. The CTDs of both enzymes are structurally unrelated, with *E. coli* TopI having Zn finger and Zn finger-like domains, and mycobacterial TopI’s C-terminal consisting of structural repeats with a unique fold [59]. *E. coli* TopI has an additional α-helix in its second Zn finger domain that interacts with the hinge region in domain DII and may be involved in DNA-gate opening regulation by pushing or pulling at the hinges [85] (Figure 4). *Sulfolobus solfataricus* TopIII has a single Zn finger of a different type on its CTD, which also contacts domain DII at the hinges, possibly with a similar role [86] (Figure 4).

The activity of topoisomerases can also be regulated by their interacting partners. Topo III enzymes are often associated with helicases that provide single-stranded DNA regions, but there is also evidence for direct regulation. In yeast, TopIII is part of a larger complex that participates in the resolution of double Holliday junctions and stalled replication forks. It consists of the Sgs1 helicase, Rmi1 protein, RPA (replication protein A), which binds single-stranded DNA, and TopIII [75]. Rmi1 was shown to redirect the preferential activity of TopIII from supercoil relaxation to decatenation, by stabilising the cleavage intermediate. Furthermore, Sgs1 and RPA seemed to additionally stimulate TopIII not just through their primary activities but also through specific interactions. In contrast with the human homologous complex of BLM helicase with TopIIIα-RMI1-RMI2, it was suggested that BLM reduces DNA-gate flexibility [83].

The crystal structure of a human TopIIIα in a complex with the interacting domain from RMI1 (OB fold) revealed that RMI1 inserts a loop into the toroid hole of the TopIIIα [70] (Figure 4). This long loop contacts the hinges in domain DII, likely directly affecting gate opening. Eukaryotic Topo III enzymes do not have a decatenation loop, and RMI1 provides this element in trans to enable efficient decatenation. This hypothesis fits rather well with the observations made on *E. coli* TopI and TopIII discussed earlier, suggesting that stabilisation of the open gate conformation is favourable for decatenation, whereas this is not required for supercoil relaxation.

Human TopIIIβ has a different interaction partner, the TDRD3 protein, which interacts with the topoisomerase in a similar way as RMI1 does with TopIIIα, but with a shorter loop [71]. TDRD3 also binds to domain DII of TopIIIβ at the same position, interacting with the hinges and possibly regulating gate opening (Figure 4). Yang et al. demonstrated that TDRD3 enhances DNA relaxation by TopIIIβ and proposed that their interaction could reduce the flexibility of the DNA-gate to facilitate T-strand passage and/or G-strand re-ligation [66].

Reverse gyrases are equipped with their own RecA helicase domain on the N-terminal end. An element called latch in the helicase domain interacts with domain DIII (CAP) of the topoisomerase to prevent the gate from opening on its own [80,81] (Figure 4). The conformational change of the helicase domain induced by the ATP binding is thought to release the latch [100]. When compared to reverse gyrase, most Topo IA enzymes have their associated domains or protein partners on the opposite side of the toroid hole. This could mean that positive supercoiling uses a different way to control how the DNA gate opens.

## 4. Conclusions

Over the past twenty years, a lot of information has been learned about how the Topo IA and IIA families simplify their DNA topology. Biochemical, kinetics, and structural biology experiments have provided some clues on how DNA cleavage and re-ligation are coupled to conformational changes of the conserved functional domains forming the DNA gate of the type IA and IIA DNA topoisomerases.

Advances in structural biology have led to the determination of several conformations of the large nucleoprotein complexes of type IIA topoisomerases, revealing some of the key allosteric mechanisms of DNA gate opening. Although the molecular basis for specific DNA topology resolution remains to be elucidated, the fact that type IIA intermolecular reactions involve solely DNA duplexes as primary substrates at the DNA gate somehow facilitates our understanding of these mechanisms.

In contrast, the diversity of substrates and reactions that can be recognised and catalysed by type IA enzymes represents an additional complexity that has to be considered for experimental designs. However, this has been partly compensated by several important studies highlighting the dynamic and kinetic properties of the type IA enzymes.

Together, more information is needed to fully understand the key steps of strand passage and the subsequent conformational changes for both families. Recent studies have highlighted that the combination of different techniques, including single-molecule approaches, is key to getting a comprehensive view of these complex mechanisms. Structural studies of the type IA and type IIA DNA topoisomerases have proven challenging, sometimes due to their relatively large size but mostly owing to their modular and highly flexible architecture. Stabilizing other reaction intermediates or building up larger DNA topology substrates to approach physiological conditions, will now be necessary to get insights into the critical steps that occur beyond the DNA gate.

## Data Availability

Data sharing not applicable.
